# Localized bioimpedance reactance as a biophysical parameter of muscle recovery following total knee arthroplasty: a prospective self-controlled study

**DOI:** 10.3389/fphys.2026.1745998

**Published:** 2026-03-24

**Authors:** Gloria Pedemonte-Parramón, Lexa Nescolarde, Ester García-Oltra, Vicente López-Pérez, Francisco Aliaga-Orduña, José A. Hernández-Hermoso

**Affiliations:** 1 Department of Orthopedic Surgery and Traumatology, Hospital Universitari Germans Trias i Pujol, Barcelona, Spain; 2 Department of Surgery, Universitat Autònoma de Barcelona, Edifici U, Campus UAB, Barcelona, Spain; 3 Department of Electronic Engineering, Universitat Politècnica de Catalunya, Barcelona, Spain

**Keywords:** bioelectrical impedance, muscle quality, muscle remodeling, quadriceps muscle, TKA postoperative recovery, TKA rehabilitation

## Abstract

**Background:**

Quadriceps weakness and atrophy are common in patients with knee osteoarthritis and can persist after total knee arthroplasty (TKA), affecting functional recovery. Localized bioelectrical impedance analysis (L-BIA) allows non-invasive assessment of muscle status through resistance (R) and reactance (Xc), reflecting muscle composition and cell integrity. This study investigated longitudinal changes in quadriceps Xc following TKA and their association with functional outcomes.

**Methods:**

Twenty-five patients undergoing unilateral TKA were evaluated preoperatively and at 6 and 12 months postoperatively. L-BIA parameters (R and Xc) were measured in the vastus medialis (VM), vastus lateralis (VL), and rectus femoris (RF). Functional outcomes were assessed using the Knee Society Score (KSS) and the Western Ontario and McMaster Universities Osteoarthritis Index (WOMAC). Interlimb comparisons and predictive performance of Xc for functional recovery were analyzed using ROC curves, Gini indices, and Kolmogorov–Smirnov statistics.

**Results:**

R values remained stable across all muscles postoperatively (P > 0.05). Xc values significantly decreased at 6 months in VM and RF: P < 0.01; VL: P = 0.04 and recovered by 12 months to pre-TKA levels, with greater percentage changes in VM and VL than RF. Also, pre-TKA, Xc was lower in the operated limb compared to the contralateral side (P < 0.01). By 12 months, Xc in VM and RF was similar between limbs, while VL remained slightly lower in the operated leg. ROC analysis showed excellent predictive performance of Xc for WOMAC outcomes (VM and VL Gini = 0.909; K-S = 0.955), with optimal cut-offs of 15.65 Ω and 14.3 Ω, respectively. Functional improvements were most pronounced in the first 6 months and correlated with Xc recovery, particularly in VM at 12 months.

**Conclusion:**

Quadriceps Xc measured by L-BIA decreases initially after TKA but recovers by 12 months, paralleling improvements in pain and function. Xc provides a sensitive, non-invasive biophysical parameter for monitoring quadriceps muscle recovery and may inform individualized rehabilitation strategies.

## 1 Introduction

Knee osteoarthritis (OA) is characterized by quadriceps weakness caused by muscle atrophy and neuromuscular activation deficits (Mizner et al., 2005a; Mizner et al., 2005b; Meier et al., 2008). These alterations, often asymmetric and accompanied by increased intramuscular fat infiltration, appear early and more pronounced in the quadriceps than in other muscle groups, leading to functional impairment and increased risk of falls (Pedroso et al., 2019; Yamauchi et al., 2019; Yamauchi et al., 2020; Paravlic et al., 2020; Okada et al., 2025).

Total knee arthroplasty (TKA) is the definitive treatment for patients with knee OA who fail conservative management (Mizner et al., 2005a; Greene and Schurman, 2008; Meier et al., 2008). However, it must address not only pre-existing quadriceps weakness, but also the additional surgical trauma to the extensor mechanism (Mizner et al., 2005a; Mizner et al., 2005b; Meier et al., 2008). This often results in early postoperative quadriceps atrophy, which has been strongly associated with delayed functional recovery (Stevens et al., 2003; Mizner et al., 2005a; Meier et al., 2008; Lastayo et al., 2009), Although pain typically decreases after TKA, up to 30% of patients experience persistent quadriceps weakness for a year or longer (Meier et al., 2008; Toth et al., 2022), largely due to ongoing atrophy and neuromuscular activation deficits (Meier et al., 2008; Meier W et al., 2009).

The extent and pattern of muscle atrophy before and after surgery, as well as the factors influencing recovery, remain poorly understood. Furthermore, there is no consensus on the most accurate and accessible method for assessing muscle atrophy and quality. The most common clinical approach—measuring thigh circumference—is simple but fails to identify which quadriceps muscles are most affected and provides no information on muscle composition. Its results are also highly dependent on measurement site, limiting reproducibility (Layec et al., 2014; Kono et al., 2025). Ultrasound and magnetic resonance imaging (MRI) can quantify muscle cross-sectional area but are expensive, time-consuming, and require specialized operators (O’Sullivan et al., 2009; Chromy et al., 2015; Kono et al., 2025). Furthermore, they are not always feasible for routine or longitudinal monitoring due to their limited accessibility. Histological analysis offers detailed insights into muscle fiber structure but is invasive and unrepresentative of overall muscle condition (Greene and Schurman, 2008; Boccaccio et al., 2021; Toth et al., 2022).

Whole-body (WB) (Lukaski, 1996) tetra-polar phase-sensitive 50 kHz bioimpedance analysis (BIA) is a commonly used non-invasive method for body composition assessment, either through prediction equations (Ellis, 2000) or Bioelectrical Impedance Vector Analysis (BIVA) (Piccoli et al., 1994). Two electrodes—one for current injection (I) and one for voltage sensing (V)—are placed on the right hand at the third metacarpophalangeal joint and the carpus, while the other pair are placed on the foot at the third metatarsophalangeal joint and the tarsus (Lukaski et al., 1986).

Resistance (R) and reactance (Xc) are the two parameters obtained directly from bioimpedance measurements. R represents the opposition to the flow of the injected alternating current, mainly through the extracellular fluid (ECF), whereas Xc represents the dielectric or capacitive component of cell membranes, organelles, and tissue interfaces (Foster and Lukaski, 1996). The phase angle (PA) relates R and Xc as PA = $\tan^{-1}=\frac{X_{c}}{R}$ and is mainly used as an indicator of malnutrition risk and prognosis in intensive care unit patients (Lukaski et al., 2017; Samoni et al., 2016).

For site-specific bioimpedance measurements, segmental-BIA (S-BIA) (Ward, 2012) and localized-BIA (L-BIA) (Nescolarde et al., 2023) have been proposed as alternative to the whole-body electrode arrangement (Kyle et al., 2004). The main difference between these techniques lies in electrode placement. In S-BIA only the voltage-sensing electrodes are repositioned to assess different body segments, while the current-injecting electrodes remain fixed at the metatarsophalangeal joint and metacarpophalangeal joint, like whole-body configuration (Chumlea et al., 1988). In contrast, in L-BIA, first proposed by Nyboer (1950), the two current-injecting (I) electrodes are placed distally, while the two voltage-sensing electrodes (V) are positioned adjacent to the specifically defined anatomical region.

Using L-BIA, Lukaski and Moore (2012) demonstrated in wound healing that changes in R were inversely proportional to ECF and directly proportional to fibrin clot formation and epithelialization, whereas increases in Xc reflected epidermal proliferation and granulation, and decreases were associated with infection and cell loss. Rutkove et al. (2008), Rutkove et al. (2017) using electrical impedance myography with arrays of needle electrodes in specific anatomical regions, identified muscle pathology in patients with neuromuscular diseases. In professional football players, L-BIA has shown a substantial reduction in Xc (by percentage of change) according to the grade of muscle injury (cell disruption) based on muscle gap (Nescolarde et al., 2013; Nescolarde et al., 2015; Nescolarde et al., 2017) and anatomical location (Nescolarde et al., 2020).

To our knowledge, this is the first study to assess the muscle status of the quadriceps muscle group (rectus femoris, vastus medialis, and vastus lateralis) before total knee arthroplasty (TKA) and at 6- and 12-month follow-ups post-TKA using localized bioelectrical impedance analysis (L-BIA), and to examine its relationship with WOMAC and KSS scores.

Given that quadriceps atrophy involves muscle fiber reduction and fat infiltration—features detectable with L-BIA—this study aimed to determine whether L-BIA can accurately assess and monitor quadriceps muscle changes before and after TKA. Expanding the use of L-BIA into orthopedic and rehabilitation settings may provide a noninvasive, objective biophysical measurement for evaluating muscle quality and recovery following TKA.

## 2 Methods

### 2.1 Study design

A prospective, longitudinal observational study with within-subject (self-controlled) comparisons study was conducted at a tertiary referral hospital between 2021 and 2023. The study was approved by the Institutional Review Board in accordance with the Declaration of Helsinki (Approval No. PI-20-045). Written informed consent was obtained from all participants prior to enrollment.

### 2.2 Patients

Twenty-five patients who underwent total knee arthroplasty (TKA) for primary osteoarthritis were included.

Inclusion criteria: age 40–85 years; diagnosis of primary knee osteoarthritis graded III or higher according to the Ahlbäck classification (Ahlbäck S, 1968); presence of intense pain and functional impairment unresponsive to nonsurgical treatment for >6 months.

Exclusion criteria: 1) cardiovascular or pulmonary disease, or high anesthetic risk; (2) polyarticular involvement of the lower-limb or lumbar spine causing functional limitation; (3) neuromuscular or neurodegenerative disorders; (4) known metal allergy requiring a specific implant; (5) withdrawal of consent.

### 2.3 Clinical assessments

Patients were evaluated preoperatively and at 6 and 12 months postoperatively.

Collected variables included age, sex, affected side, prior knee or contralateral TKA surgery, height, weight, body mass index (BMI), and American Society of Anesthesiologists (ASA) score.

Knee range of motion (ROM) was measured using a goniometer (Model 27340, Gima, Gessate, Italy). The fulcrum was aligned with the knee joint, with one arm parallel to the greater trochanter and the other to the lateral malleolus.

Clinical outcomes were assessed using the Knee Society Score (KSS) (Insall JN et al., 1989) and the Western Ontario and McMaster Universities index (WOMAC) (Bellamy N et al., 1988).

### 2.4 Surgical procedure

All surgeries were performed by senior orthopedic surgeons using the same standardized technique.

Antibiotic prophylaxis consisted of 2 g of cefazolin administered 30–60 min before surgery; penicillin-allergic patients received 900 mg of clindamycin. A tourniquet was applied in all cases.

TKA was performed using a measured resection and gap-balancing technique. Two prosthesis models were randomly assigned (Evolution™ or Columbus™). An anterior longitudinal incision with medial parapatellar arthrotomy and complete posterior cruciate ligament (PCL) resection were performed. Distal femoral cut was made using an intramedullary guide perpendicular to the mechanical axis in the coronal plane and 3° of external rotation relative to the posterior condylar axis. Proximal tibial cut used an extramedullary guide perpendicular to the mechanical axis in the coronal plane and 0°–3° posterior tibial slope. The patella was resurfaced in all cases. No drains were used.

Antithrombotic prophylaxis consisted of 40 mg of low-molecular-weight heparin daily for 30 days postoperatively.

### 2.5 Postoperative rehabilitation protocol

All patients followed a strictly standardized rehabilitation protocol established and supervised by the Department of Rehabilitation. No individualized or modified rehabilitation pathways were applied, and no variability in rehabilitation content or progression was permitted between participants.

Passive joint mobilization was initiated on the day of surgery. On postoperative day one, all patients were assisted into a seated position, performed bed-based exercises, and began ambulation in the afternoon as tolerated. Continuous passive motion (CPM) was applied using the same device (Kinetec™) according to institutional protocol.

From postoperative day two, patients attended supervised physiotherapy sessions in the hospital gymnasium. Rehabilitation focused on balance training, progressive muscle strengthening, and gait training using parallel bars. On postoperative day three, patients continued the same exercises and additionally performed stair ascent and descent training under physiotherapist supervision.

Patients were typically discharged between postoperative days three and four. After discharge, all participants followed a structured home-based rehabilitation program, delivered by physiotherapists from the same institutional rehabilitation team. Home physiotherapy sessions were conducted three to four times per week, each lasting approximately 30 min, and followed the same exercise progression as initiated during inpatient rehabilitation, including strengthening, balance, and gait training.

In addition, patients were actively encouraged to perform the prescribed exercises on days without supervised physiotherapy sessions. Adherence to unsupervised exercise practice was not objectively monitored and therefore represents the only potential source of inter-individual variability in rehabilitation exposure.

### 2.6 Localized bioimpedance measurements

**FIGURE 1:**
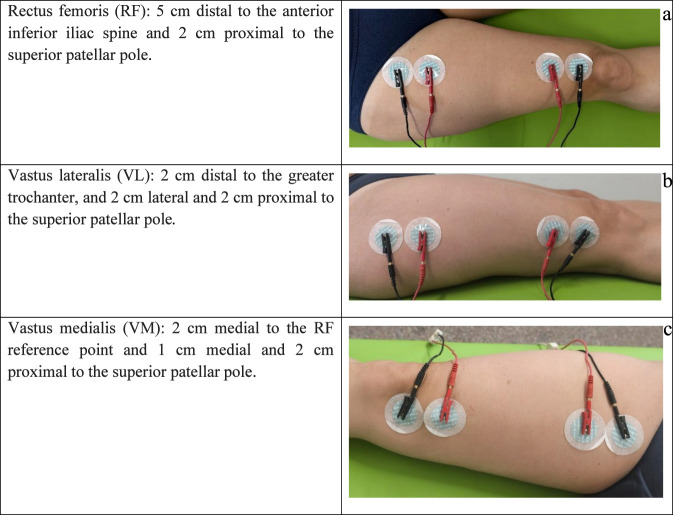
Electrode placement for quadriceps muscle groups: **(a)** rectus femoris (RF), **(b)** vastus lateralis (VL), **(c)** vastus medialis (VM).

Tetrapolar localized bioimpedance (L-BIA) was performed with patients in the supine position after a 15-min rest. Two current-injecting (black) and two voltage-sensing (red) electrodes were placed on three quadriceps muscles (Figure 1).

The voltage-electrode (V) distances were maintained for all repeated measurements to ensure consistency.

Finite element method was used to analyze sensitivity distributions simulations of surface electrode configurations (Rutkove et al., 2017).

### 2.7 Bioimpedance device

Measurements were obtained using a phase-sensitive tetrapolar impedance analyzer (BIA 101 Anniversary, Akern Srl, Florence, Italy). The instrument applied a constant sinusoidal current of 245 μA RMS at 50 kHz. The measurement range was 0–1500 Ω for resistance (R) and 0–500 Ω for reactance (Xc), with ≤2% tolerance. Measurement errors were <1 Ω for R and <2% for Xc. Disposable Ag/AgCl adhesive electrodes (Ambu® WhiteSensor Ref. CMM/1-00-S/30 Brussels, Belgium) with intrinsic R = 27.00 Ω and Xc = 0.25 Ω were used.

### 2.8 Statistical analysis

Normality of R and Xc values for the quadriceps muscle groups (rectus femoris [RF], vastus medialis [VM], and vastus lateralis [VL]) was assessed using the Shapiro–Wilk test, while homogeneity of variances was evaluated with Levene’s test. Normally distributed variables are presented as mean ± standard deviation (SD) and 95% confidence intervals (CI; lower–upper limb). Non-normally distributed variables are reported as median with interquartile range (IQR) and minimum–maximum values. Categorical variables are expressed as percentages.

Changes in the operated leg over time (1: pre-TKA; 2: 6-month post-TKA; 3: 12-month post-TKA) were analyzed using repeated-measures ANOVA and Bonferroni post-hoc test; the Friedman test was used for nonparametric data.

Percentage changes (%Δ) in localized bioimpedance analysis (L-BIA) parameters of the quadriceps muscle groups before and after TKA, expressed as median (IQR) and minimum–maximum values, were analyzed using the Wilcoxon signed-rank test.

Comparisons between the operated and non-operated limbs for L-BIA quadriceps muscle group parameters at baseline (pre-TKA) and at 12 months post-TKA were performed using paired T-tests.

Receiver operating characteristic (ROC) curves were used to evaluate the predictive ability of Xc values at 12 months for functional recovery, defined by WOMAC scores.

The effect size for the statistical test was done for each statistical test according to Serdar et al. (2021).

Sample size was estimated using MedCalc v19.0.3 (MedCalc Software, Ostend, Belgium) with α level of 0.05 and a β level of 0.20 (80% power) *a posteriori* because no previous studies have reported reference values for resistance (R) and reactance (Xc) in patients undergoing TKA at the rectus femoris, vastus medialis, and vastus lateralis. Although, previous studies in professional football player, reported minimum changes in L-BIA resistance and reactance standard deviation of the reference values (Nescolarde et al., 2015), confirming the need of the small simple size.

All analyses were performed using IBM SPSS Statistics V30.0 (Armonk, NY, United States) and statistical significance was set at *p* < 0.05.

## 3 Results

### 3.1 Demographic variables

Table 1 shows demographics variables. Parametric variables as show as mean ± standard deviation and 95% confidence interval (CI, lower-upper limb) and non-parametric variables as percentage (%).

**TABLE 1:** Demographics characteristics of the study population.

Sample size	Age (years)	BMI (kg/m^2^)	Gender	Side	ASA
N = 25	70.1 ± 6.9 (67.3–73.0)	31.7 ± 4.90 (29.6–33.7)	Female: (n = 18) 72% Male: (n = 7) 28%	Left: (n = 9) 36% Right: (n = 16) 64%	I: (n = 1) 4% II: (n = 21) 84% III: (n = 3) 12%

ASA: american society of anesthesiologists.

### 3.2 L-BIA parameters

R values in the VM, VL, and RF showed no significant changes after TKA compared with pre-TKA measurements (P > 0.05). In contrast, Xc values in all three muscles decreased significantly at 6 months post-TKA (RF and VM: P < 0.01; VL: P = 0.04). Between 6 and 12 months, Xc values progressively increased, returning to levels comparable to those observed preoperatively (Table 2).

**TABLE 2:** L-BIA parameters of Quadriceps Muscle Groups Pre- and Post- TKA surgery.

Quadriceps muscle groups	L-BIA	1-week pre-TKA^1^	6-month post-TKA^2^	12-month post-TKA^3^	P^A^ Ω^2^	P^1-2^_r_	P^2-3^_r_	P^1-3^_r_
Rectus femoris (RF)	R (Ω)	125.0 ± 31.8 (112.0–138.0)	115.0 ± 24.0 (105.0–125.0)	122.0 ± 29.0 (110.0–135.0)	0.17 0.08	0.22 0.37	0.75 0.24	1.00 0.18
	Xc (Ω)	7.3 ± 2.1 (6.5–8.1)	6.6 ± 1.9 (5.8–7.3)	7.8 ± 2.1 (6.9–8.6)	<0.01* 0.22	0.02* 0.53	0.01* 0.57	0.49 0.29
Vastus medialis (VM)	R (Ω)	129.1 ± 34.9 (114.7–143.5)	129.3 ± 32.2 (116.0–142.6)	130.4 ± 32.4 (116.4–144.4)	0.93 0.003	1.00 0.06	1.00 0.07	1.00 0.03
	Xc (Ω)	8.0 ± 2.4 (7.0–8.9)	7.0 ± 2.1 (6.1–7.9)	7.8 ± 2.7 (7.1–9.8)	<0.01* 0.22	0.04* 0.50	0.01* 0.56	0.26 0.35
Vastus lateralis (VL)	R (Ω)	122.1 ± 34.7 (107.8–136.4)	120.2 ± 26.1 (109.5–131.0)	124.5 ± 32.3 (110.5–138.4)	0.91 0.004	1.00 0.08	1.00 0.09	1.00 0.001
	Xc (Ω)	9.0 ± 2.9 (7.9–10.2)	7.7 ± 2.3 (6.8–8.6)	9.2 ± 3.9 (7.6–10.8)	0.04* 0.13	<0.01* 0.68	0.09 0.44	1.00 0.10

3 L-BIA, quadrceps muscle groups pre- and post- Total Knee Arthroplasty (TKA) surgery as mean ± standard deviation and 95% CI (lower-upper limb). R: resistance; Xc: reactance; P^A^, from ANOVA, repeated measurements test; Ω2: effect size for ANOVA, small size; P1-2, P2-3 and P1- post-hoc Bonferroni analysis; r: effect size for post-hoc Bonferroni test.

The percentage change in Xc over time (Table 3) was greater in all muscle groups analyzed (VM, RF, and VL). However, the largest and most significant increases were observed in the VM and RF muscles.

**TABLE 3:** Percentage of change in L-BIA quadriceps muscle groups repeated measurements.

Quadriceps muscle groups	L-BIA	% Δ (2-1)	% Δ (3-1)	P^W^_r_
Rectus femoris (RF)	R (Ω)	−7.4 (20.1) [(-36) – 29.7]	1.1 (27.8) [(-36.5) – 37.3]	0.26 −0.28
	Xc (Ω)	−11.2 (20.7) [(-38.7) – 28.9]	1.4 (29.1) [(-100.0) – 137.8]	<0.01* −0.63
Vastus medialis (VM)	R (Ω)	2.5 (22.2) [(-26.9) – 39.6]	−1.5 (22.2) [(-34.1) – 59.2]	0.96 −0.01
	Xc (Ω)	−16.67 (32.3) [(-40.0) – 44.0]	8.70 (39.0) [(-42.9) – 185.5)]	<0.01* −0.77
Vastus lateralis (VL)	R (Ω)	0.1 (22.9) [(-31.2) – 47.4]	2.4 (16.8) [(-35.2) – 41.6)]	0.62 −0.12
	Xc (Ω)	−15.3 (19.7) [(-40.0) – 31.1]	−3.8 (21.5) [(-30.3) – 262.3]	0.02* −0.57

L-BIA, percentage of change (% Δ) quadriceps muscle groups pre- and post- Total Knee Arthroplasty (TKA) surgery expressed as median and IQR (interquartile range) and minimum-maximum values. 1: pre-TKA, 1-week; 2: 6-month post-TKA; 3: 12-month post-TKA.

R: resistance; Xc: reactance; PW < 0.05 from Wilcoxon test; r: effect size for Wilcoxon test.

### 3.3 Interlimb comparisons

Preoperatively, Xc values in all quadriceps muscles were significantly higher in the non-operated limb compared to the operated side (P < 0.01). At 12 months after-TKA, Xc values in the RF and VM became comparable between limbs (P > 0.05), while the VL remained significantly higher in the non-operated leg (P < 0.01). No significant interlimb differences in R values were observed either pre-TKA or at 12 months post-TKA (Table 4).

**TABLE 4:** L-BIA quadriceps muscle groups pre- and 12 months post-TKA surgery: comparison between operated and non-operated extremity.

		Pre-TKA			12-month post-TKA		
Quadriceps muscle groups	L-BIA	Operated	Non-operated	P^T^ Cohen’s d	Operated extremity	Non-operated extremity	P^T^ Cohen’s d
Rectus femoris (RF)	R (Ω)	125.0 ± 31.8 (112.0–138.0)	113.5 ± 43.0 (96.5–130.5)	0.34 0.19	122.0 ± 29.0 (110.0–135.0)	125.1 ± 29.4 (112.4–137.8)	0.47 −0.18
	Xc (Ω)	7.3 ± 2.1 (6.5–8.1)	8.4 ± 2.3 (7.5–9.4)	<0.01* −0.72	7.8 ± 2.1 (6.9–8.6)	7.9 ± 1.90 (7.1–8.7)	0.62 0.10
Vastus medialis (VM)	R (Ω)	129.1 ± 34.9 (114.7–143.5)	131.5 ± 31.6 (118.5–144.6)	0.35 −0.19	130.4 ± 32.4 (116.4–144.4)	132.3 ± 36.1 (116.7–148.0)	0.52 −0.14
	Xc (Ω)	8.0 ± 2.4 (7.0–8.9)	9.1 ± 2.6 (8.0–10.2)	<0.01* −0.64	9.0 ± 3.5 (7.6–10.4)	9.1 ± 2.7 (7.9–10.3)	0.71 0.08
Vastus lateralis (VL)	R (Ω)	122.1 ± 34.7 (107.8–136.4)	121.1 ± 32.1 (107.8–134.43)	0.60 0.11	124.5 ± 32.3 (110.5–138.4)	123.8 ± 30.8 (110.5–137.1)	0.79 0.06
	Xc (Ω)	9.0 ± 2.9 (7.9–10.2)	10.2 ± 2.7 (9.1–11.3)	<0.01* −0.70	9.2 ± 3.9 (7.6–10.8)	10.3 ± 4.4 (8.4–12.2)	0.04* −0.45

L-BIA, quadriceps muscle groups pre- and post- Total Knee Arthroplasty (TKA) surgery as mean ± standard deviation and 95% CI (lower-upper limb). P^T^, for t-test; Cohen’s d: effect size for t-test.

### 3.4 Functional outcomes

Both the KSS and WOMAC scores improved significantly at 6 and 12 months post-TKA compared to pre-TKA values (P < 0.01). The greatest improvement occurred during the first 6 months. Between 6 and 12 months, no further significant changes were observed, except for the WOMAC stiffness subscale, which continued to improve at 12 months (P < 0.01) (Table 5).

**TABLE 5:** KSS and WOMAC scores result pre- and post-TKA (6- and 12-month).

Clinical score		1-week pre-TKA^1^	6-month post-TKA^2^	12-month post-TKA^3^	P^F^ Kendall’s W	P^1-2^_r_	P^2-3^_r_	P^1-3^_r_
KSS	Total	105 (29) (70–137)	162 (30) (89–192)	166 (25.5) (70–199)	<0.01 0.69	<0.01 −0.99	0.31 −0.25	<0.01 −0.96
	Knee	59 (16) (22–68)	84 (17) (44–99)	86 (19) (45–100)	<0.01 0.61	<0.01 −0.95	0.28 −0.27	<0.01 −0.96
	Function	50 (15) (20–70)	80 (20) (45–100)	85 (20) (25–100)	<0.01 0.68	<0.01 −1.00	0.74 −0.10	<0.01 −0.98
WOMAC	Total	53 (19) (24–72)	20 (15) (0–45)	9 (18) (0–41)	<0.01 0.78	<0.01 1.00	0.02 0.59	<0.01 1.00
	Pain	10 (2) (6–15)	4 (5) (0–11)	3 (3.5) (0–9)	<0.01 0.63	<0.01 0.98	0.06 0.48	<0.01 1.00
	Stiffness	4 (2) (1–7)	2 (3) (0–6)	1 (2) (0–4)	<0.01 0.39	<0.01 0.66	<0.01 0.84	<0.01 0.98
	Function	38 (12) (14–55)	13 (9.5) (1–31)	8 (12) (0–31)	<0.01 0.68	<0.01 1.00	0.13 0.39	<0.01 1.00

Median and IQR (interquartile range) and minimum-maximum values. P^F^, from Friedman test for repeated measurements; Kendall’s W: effect size for Friedman test; P^1-2^. P^2-3^and P^1-3^. Wilcoxon test; r: effect size for Wilcoxon test.

### 3.5 Predictive performance of Xc parameters

**FIGURE 2:**
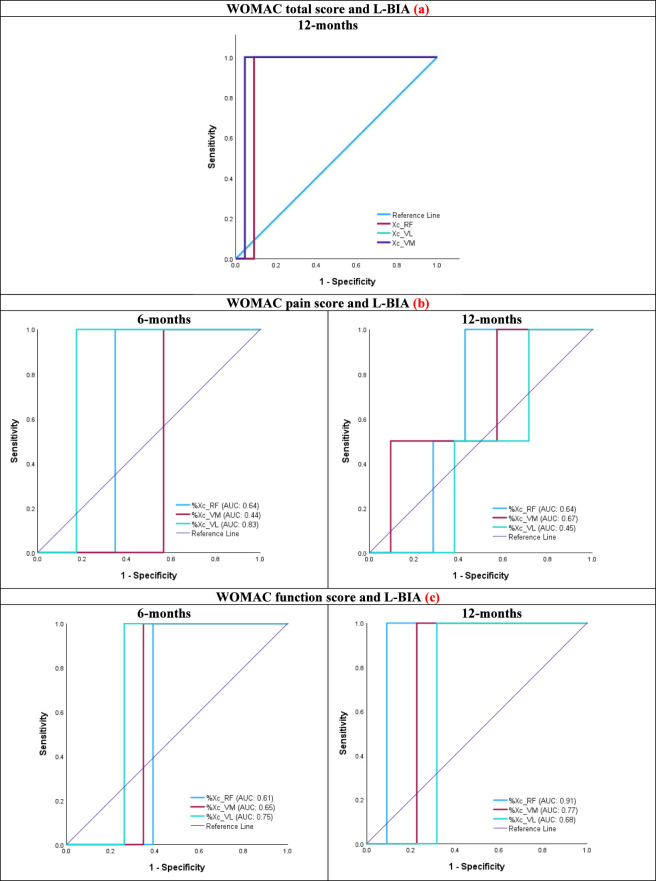
ROC curve: **(a)** WOMAC Total score in relation to Xc L-BIA post-TKA at rectus femoris (RF), vastus lateralis (VL), and vastus medialis (VM) 12-month post-TKA surgery, **(b)** WOMAC pain score in relation to Xc L-BIA percentage of change (%Δ Xc) at 6- and 12-month post-TKA surgery at RF, VL, and VM, and **(c)** WOMAC function score in relation to Xc L-BIA percentage of change (%Δ Xc) at 6- and 12-month post-TKA surgery at RF, VL, and VM.

ROC curve analysis at 12 months post-TKA demonstrated excellent predictive ability of Xc values for functional outcomes measured by the WOMAC score. Gini indices ranged from 0.818 to 0.909, and Kolmogorov–Smirnov (K-S) statistics from 0.909 to 0.955. The highest predictive performance was observed in the VM and VL, both showing Gini values of 0.909 and K-S statistics of 0.955. The optimal Xc cut-off values for predicting better WOMAC outcomes 15.65 Ω for the VM and 14.3 Ω for the VL (Table 6; Figure 2a).

**TABLE 6:** ROC curve metrics between WOMAC and Xc values at 12 months post-TKA.

L-BIA 12-month Post-TKA	AUC	S. E	Asymptotic	Asymptotic 95% CI		Gini index	K-S statistics	
				Lower bound	Upper bound		Max K-S^a^	Cut-off^b^ (Ω)
Xc_ RF	0.909	0.061	0.00	0.789	1.029	0.818	0.909	10.55
Xc_ VM	0.955	0.044	0.00	0.868	1.042	0.909	0.955	15.65
Xc_VL	0.955	0.044	0.00	0.868	1.042	0.909	0.955	14.30

AUC: area under the curve; SE: standard error; K-S: Kolmogorov-Smirnov. a: The maximum Kolmogorov-Smirnov (K-S) metric. Also, the maximum value of Youden’s index; b: In case of multiple cutoff values associated with Max K-S, the largest one is reported.

### 3.6 Discriminative capacity of Xc in relation to WOMAC subscales

At 6 months postoperatively, L-BIA parameters demonstrated the strongest relationship with the WOMAC pain subscale, primarily driven by improvements in the VL and RF (Figure 2b). By 12 months, only the VM showed further increase in its association with pain, while VL and RF remained stable or slight decline.

Regarding the WOMAC function subscale, correlations with L-BIA parameters were moderate at 6 months but became stronger at 12 months (Figure 2c). Initially, the VL exhibited the most notable association with function; however, by 12 months, VM and RF exhibited stronger correlations, indicating a progressive shift in muscle contribution to functional recovery over time.

## 4 Discussion

This study evaluated the use of localized bioimpedance analysis (L-BIA) as a novel, noninvasive tool for assessing quadriceps muscle atrophy and recovery following total knee arthroplasty (TKA). The principal finding was that after-TKA reactance (Xc) values of all quadriceps muscles decreased up to 6 months postoperatively—suggesting muscle atrophy—and subsequently increased to preoperative or higher levels by 12 months, paralleling improvements in pain and function. These results suggest that L-BIA can sensitively detect structural muscle changes and reflect postoperative recovery dynamics.

### 4.1 Quadriceps atrophy and preoperative asymmetry

Quadriceps weakness and atrophy are well-recognized sequelae of knee osteoarthritis (OA) and TKA (Mizner et al., 2005a; Mizner et al., 2005b; Meier et al., 2008). While muscle strength and neuromuscular activation have been widely studied, the structural and compositional state of the quadriceps has received less attention. Quadriceps weakness and atrophy are typically more pronounced in the painful limb but may also occur in the contralateral leg, since patients with knee OA usually exhibit bilateral deficits compared with healthy individuals of the same age. This asymmetry often persists even after surgery (Mizner et al., 2005a; Meier et al., 2008; Meier W et al., 2009; Moon et al., 2016; Singla et al., 2023).

In our study, preoperative Xc values were lower in the painful leg awaiting TKA, assuming the presence of quadriceps muscle atrophy. In contrast, the contralateral, non-operated limb displayed higher and more stable Xc values during follow-up. However, these results were not compared with an age-matched healthy population, and thus the true magnitude of atrophy cannot be precisely quantified.

### 4.2 Postoperative evolution of quadriceps muscles

Following total knee arthroplasty, our results demonstrated a significant decline in reactance (Xc) values across all quadriceps components at 6 months, with a greater relative reduction in the vastus medialis (VM) and vastus lateralis (VL) compared with the rectus femoris (RF). These findings suggest a more pronounced functional or structural impairment of the VM and VL during the early postoperative period. The presence of significant changes in reactance without parallel alterations in resistance indicates that post-TKA muscle impairment primarily reflects alterations in cellular integrity and tissue organization, potentially related to muscle disuse, denervation, or structural remodelling, rather than changes in extracellular fluid content.

Previous studies have consistently reported that quadriceps weakness and muscle atrophy worsen in the initial months after TKA (Mizner et al., 2005b; Meier et al., 2008; Lastayo et al., 2009; Petterson et al., 2011; Singla et al., 2023), with differential involvement of individual muscle components. In particular, VM and VL appear to be more affected than RF (Miokovic et al., 2012; Toth et al., 2022), a pattern commonly attributed to their greater recruitment during functional tasks such as standing and gait. Although these studies primarily assess muscle strength or morphology, this distribution aligns with the muscle-specific Xc changes observed in our cohort, supporting the physiological plausibility of our findings.

Regarding recovery, our data indicate that quadriceps muscle recovery, as reflected by Xc values, begins around 6 months postoperatively and becomes evident by 12 months, when interlimb differences were no longer statistically significant. This temporal pattern is consistent with reports describing recovery at approximately 6 months (Lastayo et al., 2009; Moon et al., 2016; Singla et al., 2023), although later recovery trajectories have also been described (Yoshida et al., 2008; Petterson et al., 2011). Importantly, most previous studies rely on strength-based assessments and heterogeneous methodologies, which limits direct comparability. In contrast, our results provide muscle-specific, localized information based on electrical properties, offering complementary insight into quadriceps recovery after TKA.

### 4.3 Relationship between quadriceps recovery and function

Quadriceps weakness and atrophy have been closely linked to functional recovery after TKA (Mizner et al., 2005a; Meier et al., 2008; Yoshida et al., 2008; Toth et al., 2022), due to their influence on knee kinematics and stability. Patients with well-functioning quadriceps show improved gait, better knee flexion, and enhanced ability to climb stairs and perform daily activities, resulting in higher functional scores (Greene and Schurman, 2008; García-Sanz et al., 2025).

Although pain typically improves shortly after surgery (Yoshida et al., 2008), functional recovery often progresses more slowly. Up to 30% of patients fail to regain quadriceps function comparable to age-matched controls, with persistent weakness extending beyond 1 year (Meier et al., 2008; Yoshida et al., 2008; Toth et al., 2022). In our cohort, preoperative pain and functional limitations were marked. Pain decreased rapidly after surgery, reaching a plateau by 12 months, while functional improvement was modest at 6 months and more pronounced by 12 months. This delay in function parallels the gradual rise in Xc, which likely reflects regeneration and hypertrophy of muscle fibers (Nescolarde et al., 2013; Nescolarde et al., 2015).

### 4.4 Predictive value of L-BIA parameters

Analysis of L-BIA parameters revealed a strong association between Xc and postoperative WOMAC scores. ROC curve analysis demonstrated excellent discriminative capacity of Xc values for predicting functional outcomes, particularly for the VM and VL, with high Gini and Kolmogorov–Smirnov indices. These findings suggest that Xc, a bioelectrical marker of cell membrane integrity and muscle function (Talluri, 1998; Lukaski et al., 2019), may serve as a reliable predictor of clinical recovery following TKA.

When WOMAC subscales were considered, the relationship between L-BIA parameters and the pain component was strongest at 6 months, mainly through improvements in the VL and RF. By 12 months, only the VM showed further improvement, while VL and RF remained stable or slightly declined. Regarding function, correlations with L-BIA parameters were moderate at 6 months but stronger at twelve, reflecting a shift from early lateral muscle recovery to later medial muscle reactivation. This evolution suggests that as the VM regains integrity, it plays a central role in restoring knee stability and overall function.

The predominance of VM involvement aligns with its vulnerability in both OA and TKA, especially when a medial parapatellar approach is used (Meier et al., 2008; Paravlic et al., 2020). Previous research has linked persistent VM weakness to anterior knee pain, instability, and poorer functional outcomes (Chester et al., 2008; Martinus Breugem and Haverkamp, 2014; Kim et al., 2021; Lee et al., 2021; Jung et al., 2022). We therefore hypothesize that, although the VM is the most atrophic muscle preoperatively and the slowest to recover, successful restoration of its bioelectrical integrity is closely associated with improved clinical results. In contrast, the VL tends to recover earlier and stabilize once the VM regains strength, contributing to better muscle balance and overall knee function (Miokovic et al., 2012; Martinus Breugem and Haverkamp, 2014; Boccaccio et al., 2021; Toth et al., 2022).

### 4.5 Clinical implications

The observation that quadriceps status at 12 months remains similar to preoperative levels, despite rehabilitation, is noteworthy. This may reflect the chronic and partially irreversible nature of long-standing muscle atrophy in OA. Although rehabilitation promotes recovery, it may not fully reverse chronic degeneration. Furthermore, incomplete neuromuscular recovery may contribute to persistent weakness (Lastayo et al., 2009; Meier W et al., 2009; Singla et al., 2023).

Our findings highlight the potential of L-BIA as a practical and objective method for monitoring quadriceps evolution and recovery. By quantifying muscle reactance, L-BIA could allow clinicians to tailor rehabilitation protocols to individual patients, identify those with persistent atrophy, and detect asymmetries that may lead to functional limitations, reduced knee flexion, or altered gait (Yoshida et al., 2008; García-Sanz et al., 2025). In cases where patients show persistently low Xc values, L-BIA could guide early therapeutic interventions to enhance recovery.

In addition, preoperative L-BIA screening could identify patients with significant quadriceps atrophy who might benefit from targeted prehabilitation programs to optimize postoperative outcomes. Integration of L-BIA into portable or wearable devices could also enable continuous home-based monitoring, reducing the need for frequent clinical visits and facilitating personalized rehabilitation.

### 4.6 Limitations

The use of two different prosthetic models could introduce slight biomechanical variability. However, the objective was to assess the applicability of the L-BIA system in TKA patients, which was successfully achieved. Furthermore, the contralateral limb was used as an internal control. While OA frequently affects both knees and the “healthy” limb may not be entirely normal (Meier et al., 2008), intra-individual comparison reduces confounding by sex, age, and lifestyle factors, making the results more clinically relevant. Finally, muscle atrophy cannot be detected directly on a weight-bearing X-ray. A weight-bearing radiograph is designed to assess bone and joint structures, not muscle tissue. However, chronic joint degeneration and altered alignment seen on weight-bearing X-ray may suggest reduced loading or disuse, which can contribute to muscle atrophy.

### 4.7 Conclusions

L-BIA is a novel, non-invasive, and sensitive method for evaluating quadriceps atrophy and monitoring its progression in patients undergoing TKA. Changes in reactance closely mirror clinical recovery and correlate with functional outcomes. The technique holds promise for identifying patients at risk of delayed rehabilitation and for guiding individualized pre- and postoperative management strategies.

## Data Availability

The raw data supporting the conclusions of this article will be made available by the authors, without undue reservation.
